# Methanotrophy Alleviates Nitrogen Constraint of Carbon Turnover by Rice Root-Associated Microbiomes

**DOI:** 10.3389/fmicb.2022.885087

**Published:** 2022-05-18

**Authors:** Weiwei Cao, Yuanfeng Cai, Zhihua Bao, Shuwei Wang, Xiaoyuan Yan, Zhongjun Jia

**Affiliations:** ^1^State Key Laboratory of Soil and Sustainable Agriculture, Institute of Soil Science, Chinese Academy of Sciences, Nanjing, China; ^2^University of Chinese Academy of Sciences, Beijing, China; ^3^Ministry of Education Key Laboratory of Ecology and Resource Use of the Mongolian Plateau & Inner Mongolia Key Laboratory of Grassland Ecology, School of Ecology and Environment, Inner Mongolia University, Hohhot, China; ^4^Inner Mongolia Key Laboratory of Environmental Pollution Control and Waste Resource Reuse, Inner Mongolia University, Hohhot, China

**Keywords:** methanotrophs, carbon and nitrogen flow, methane, nitrogen fixation, stable isotope probing

## Abstract

The bioavailability of nitrogen constrains primary productivity, and ecosystem stoichiometry implies stimulation of N_2_ fixation in association with carbon sequestration in hotspots such as paddy soils. In this study, we show that N_2_ fixation was triggered by methane oxidation and the methanotrophs serve as microbial engines driving the turnover of carbon and nitrogen in rice roots. ^15^N_2_-stable isotope probing showed that N_2_-fixing activity was stimulated 160-fold by CH_4_ oxidation from 0.27 to 43.3 μmol N g^–1^ dry weight root biomass, and approximately 42.5% of the fixed N existed in the form of ^15^N-NH_4_^+^ through microbial mineralization. Nitrate amendment almost completely abolished N_2_ fixation. Ecophysiology flux measurement indicated that methane oxidation-induced N_2_ fixation contributed only 1.9% of total nitrogen, whereas methanotrophy-primed mineralization accounted for 21.7% of total nitrogen to facilitate root carbon turnover. DNA-based stable isotope probing further indicated that gammaproteobacterial *Methylomona*s-like methanotrophs dominated N_2_ fixation in CH_4_-consuming roots, whereas nitrate addition resulted in the shift of the active population to alphaproteobacterial *Methylocystis*-like methanotrophs. Co-occurring pattern analysis of active microbial community further suggested that a number of keystone taxa could have played a major role in nitrogen acquisition through root decomposition and N_2_ fixation to facilitate nutrient cycling while maintaining soil productivity. This study thus highlights the importance of root-associated methanotrophs as both biofilters of greenhouse gas methane and microbial engines of bioavailable nitrogen for rice growth.

## Introduction

Rice roots serve as one of the major carbon inputs in paddy fields, also playing important roles in maintaining nutrient availability and crop yields through microbial recycling (Witt et al., 2000). It has been estimated that plant-associated matter could contribute 1,700–3,470 kg organic carbon ha^–1^, of which 12–14% could be of roots origin (Kimura et al., 2004). Root-derived soil organic carbon (SOC) is often considered more stable than that from aboveground plant residues (Gale and Cambardella, 2000; Rasse et al., 2005; Kätterer et al., 2011). In the past decade, numerous studies have focused on the influence of plant carbon input on the native microbial biomass carbon (MBC) and SOC sequestration (Lu et al., 2003; Guenet et al., 2010; Ge et al., 2012), particularly the dynamic changes of chemical composition (e.g., lignin, nonstructural carbohydrates) during rice root decomposition (Lu et al., 2010; Park et al., 2020). However, microbial mechanisms underlying the coupling of carbon and nitrogen are poorly understood, though most studies have been done with respect to the root organic carbon (ROC) allocation (CO_2_, dissolved organic carbon, SOC, MBC) or the root-derived nitrogen release (NH_4_^+^, NO_3_^–^, dissolved organic nitrogen, gas N).

Plant-microbe interactions are crucial for plant growth, productivity, phytoremediation, and the soil carbon sequestration (Bulgarelli et al., 2012; Lundberg et al., 2012; Trivedi et al., 2013). Root-associated microbes catalyze nutrient cycling (C, N, P, S, Fe) (Baya et al., 1981; Scheid et al., 2004) and play a key role on plant growth (Ding et al., 2019; Moreau et al., 2019). For instance, plant growth-promoting bacteria include *Clostridium*, Bacteroidetes (Rui et al., 2009), Proteobacteria, Sphingobacteria, and Actinobacteria (Li et al., 2011; Guo et al., 2020). Nevertheless, culture-dependent technique has significantly hampered taxonomic identification of microorganisms in association with root decomposition (Kimura et al., 1989; Murakami et al., 1990). The rapid advance of high-throughput sequencing in combination with stable isotope probing (SIP) thus provides powerful tools in recent years to establish direct links of carbon and nitrogen flows to phylogenetic identities of plant-associated microbial communities.

Rice paddies contribute 10–25% of global CH_4_ emissions, and up to 90% of the methane could be consumed by methane-oxidizing bacteria (MOB, methanotrophs) in rice roots due to radial oxygen loss (Holzapfel-Pschorn et al., 1986). The vast amount of methane oxidation in rice roots could intensify methane-driven carbon metabolism at multitrophic levels. For example, methylotrophic Hyphomicrobiaceae and phylogenetically distinct guilds of nitrogen-fixing *Rhizobia* have been revealed as key players driving CH_4_-derived carbon transfer (Murase and Frenzel, 2007; Lee et al., 2021). Moreover, plant residues are often characterized with the exceptionally high C/N ratios, leading to severe deficiency of nitrogen for microbial growth (Zechmeister-Boltenstern et al., 2015). For example, soil microbial biomass often had a value of C:N ratios about 8–9 (Cleveland and Liptzin, 2007), while rice root could be as high as 27 (Lu et al., 2010). Plant root-associated microbes could have thus likely evolved N_2_-fixing function to meet N demand. The ability to fix N_2_ in methanotrophs has long been demonstrated in pure cultures (Davis et al., 1964; Murrell and Dalton, 1983), and molecular analysis revealed distinct evolutionary trajectories of nitrogenase genes for MOB (Auman et al., 2001; Dedysh et al., 2004). A rough estimate has suggested that methanotrophy could have likely contributed 33–47% of reactive N input in water-submerged peatlands (Larmola et al., 2014), whereas our previous study showed 11.5% of plant N in rice fields was from MOB on the basis of nitrogen dilution pool extrapolation (Bao et al., 2014; Minamisawa et al., 2016). However, N_2_ fixation occurred most likely in association with root decomposition and respiration to relieve environmental stresses in complex environment (Fog, 1988; Ramirez et al., 2012; Finn et al., 2015), and the solid evidence for methanotrophy-induced carbon and nitrogen coupling is still missing.

SIP is a powerful mean to trace carbon and nitrogen flow, and to establish direct link of ecologically important processes to physiologically active taxa in complex environment (Dumont and Murrell, 2005; Lu et al., 2006). Here, to investigate the influence of methane oxidation on root carbon and nitrogen turnover and identify the active microorganisms, dual DNA-SIP (^13^CH_4_ and ^15^N_2_) was conducted. The rice roots were collected at the seedling stage and incubated in N-free or N-replete liquid medium with ^13^CH_4_ and ^15^N_2_. Root decomposition and microbial metabolism were thoroughly determined by the root biomass, microbial biomass, gases production, and dissolved compounds, respectively.

## Materials and Methods

### Rice Root Sampling and Pretreatment

Rice samples at the seedling stage (30 days after sowing in the field) were collected from the Changshu Agro-Ecological Experimental Station of the Chinese Academy of Sciences, Jiangsu Province, China (31.5497N, 120.6984E). Seeds were pre-germinated 48 h and sown into the field in waterlogged condition from May 1 to June 6, 2019. The rice seedlings were fertilized with urea, calcium superphosphate, and potassium chloride in 433.9, 535.7, and 133.9 kg ha^–2^, respectively, on May 15, supplemented by 112.5 kg ha^–2^ of urea on May 24, and 187.5 kg ha^–2^ of urea on June 2. The entire rice plants were brought back to laboratory in cold storage. After washing the loosely adherent soil particles with tap water by gentle shaking, the roots were cut off and put into 50 ml centrifuge tubes with 40 ml deionized sterile water, followed by vortex for 10 min and centrifugation at 5,000 rpm for 10 min. The fresh roots were then transferred to a new 50 ml centrifuge tube with 40 ml deionized sterile water, subjected to sonication at 40 Hz for 30 s, and centrifuged again at 5,000 rpm for 10 min to collect root pellet. The sonication and centrifugation procedures were repeated twice to ensure the complete removal of soils on the roots as previously described (Bao et al., 2014; Edwards et al., 2015). The soil-free roots were cut into 1–2 cm short pieces and divided into two subsamples. One subsample was used for microcosm incubation, whereas the other was frozen at –80°C for DNA extraction to investigate the root-associated microbial communities.

### Microcosm Construction for ^13^CH_4_ and ^15^N_2_ Stable Isotope Probing

We set three treatments to incubate the rice roots in nitrate mineral salts (NMS) or nitrate-free mineral salts (NFMS) media (Whittenbury et al., 1970) to enhance microbial growth. In brief, 1 g of fresh roots was placed into a 120-ml serum bottle amended with 40 ml medium. Treatment NoCH_4_: NFMS medium+10% ^15^N_2_+10% O_2_; treatment ^13^CH_4_: NFMS medium+10% ^13^CH_4_+10% ^15^N_2_+10% O_2_; and treatment ^13^CH_4_+NO_3_^–^: NMS medium+10% ^13^CH_4_+10% ^15^N_2_+20% O_2_. One atmosphere pressure was achieved for all serum bottles using inert argon gas. The labeled gas was purchased from the Cambridge Isotope Laboratories company (99% ^15^N-labeled for ^15^N_2_ and 99% ^13^C-labeled for ^13^CH_4_). The headspace gases were evacuated and flushed with argon gas before microcosm construction, and the serum bottles were sealed with rubber stoppers and aluminum caps. All treatments were conducted in triplicate bottles in a shaker with 150 rpm at 30°C for 30 days in dark.

### Measurement of Carbon and Nitrogen Turnover in Rice Root

#### Microbial-Respired CO_2_ From Root Organic Carbon Decomposition and CH_4_ Oxidation

Headspace gases (1 ml) in the serum bottles were sampled on days 0 and 30, respectively, for the determination of the CO_2_ emission during microcosm incubation. It includes root-derived CO_2_ and ^13^CH_4_-derived ^13^CO_2_ in treatments with CH_4_ amended. Concentrations of CO_2_ were measured using a gas chromatography (Agilent 7890, United States) equipped with a Porapak Q column and a thermal conductivity detector with N_2_ as the carrier gas. The column temperature was set at 60°C, and the detector and injector were set at 150°C. The isotope excess of ^13^C-CO_2_ was measured using a MAT253 stable isographic quality spectrometer (Thermo Fisher Scientific, Dreieich, Germany).

#### Inorganic N Species (NH_4_^+^, NO_3_^–^ and NO_2_^–^)

After microcosm incubation, all of the roots and medium in the serum bottles were transferred to 50 ml centrifuge tubes and were centrifuged at 5,000 rpm for 10 min to collect roots, as well as microbial biomass (undissolved). The supernatants (dissolved compounds) were collected for measurement of inorganic nitrogen, including NH_4_^+^, NO_3_^–^, and NO_2_^–^. The concentrations were determined by a continuous flow analytical system (San++System, Skalar, Holland), and the ^15^N isotope excess was determined using IsoPrime 100 stable isotope ratio mass spectrometer (Elementar, Germany). Nitrite was always below the detection limit for all treatments, except for the ^13^CH_4_+NO${}_{3}^{-}$ treatment, whereas ammonium was detected in all the treatments.

#### Total Organic Carbon and Total Organic Nitrogen

The root system pellets after centrifugation as mentioned above were thoroughly rinsed to remove the dissolved carbon and nitrogen (DOC, DON, and inorganic N species). The rinsed pellets were then ground into powders in liquid nitrogen. Approximately 0.2 g powders were set apart for DNA extraction, whereas the rest of the pellet powders were completely freeze-dried by Freeze Dryers (Alpha 1-2 LD plus, CHIRIST, Germany). The dry powders were passed through a sieve with 0.15 mm pore size for the determination of Total Organic Carbon (TOC) and Total Organic Nitrogen (TON) by FlashSmart Elemental Analyzer (Thermo Fisher Scientific, Dreieich, Germany). It should be noted that DOC was calculated as the difference between the undissolved TOC of root system at day 0 and CO_2_ plus undissolved TOC at day 30, and DON as the difference between undissolved TON of root system at day 0 and inorganic nitrogen plus undissolved TON at day 30. The isotope excess of ^13^C-TOC and ^15^N-TON was analyzed by Flash 2000 elemental analyzer coupled to a Delta V Advantage isotope ratio mass spectrometer (Thermo Fisher Scientific, Dreieich, Germany).

### Ecophysiology Flux Calculation of Methanotrophy-Directed Carbon and Nitrogen Metabolism in CH_4_-Consuming Rice Root

#### Flux of ^13^CH_4_ Consumption (T_CH4_)

Total CH_4_ consumption includes ^13^CH_4_-derived biomass in root system TOC, ^13^CO_2_ production in the headspace, and the ^13^C-dissolved organic carbon in liquid medium, including cell lysis, extracellular polymers (EPS), and intermediates. It was calculated as follows:


(1) $$T_{C\,H\,4}=\frac{A_{T\,O\,C}\times C_{T\,O\,C}\times 10^{6}}{M}+\frac{A_{C\,O\,2}\times C_{C\,O\,2}\times V_{g\,a\,s}}{m}$$

where T_CH4_ is total CH_4_ consumption, expressed as μmol C g^–1^ *dry weight biomass*; *A*_TOC_, atom excess of ^13^C in TOC, %; *C*_TOC_, TOC content, %; *A*_CO2_, atom excess of ^13^C in CO_2_, %; *C*_CO2_, the detected concentration of the total CO_2_ emission, μmol l^–1^; *V*_gas_, the volume of the headspace gas of the incubation bottles (80 ml, 0.08 l); *M*, molar mass of ^13^C, g mol^–1^; and *m*, the dry weight biomass of root pellets, g. It should be noted that ^13^C-dissolved organic carbon was not taken into account in our results, whereas its proportion was relatively low (<5%) as previously reported (He et al., 2020).

#### Microbial Carbon Utilization Efficiency of CH_4_-Derived C

Carbon utilization efficiency (CUE) during methanotrophy is defined as the production of ^13^C-organic carbon per mole of CH_4_ oxidized (Trimmer et al., 2015), which is calculated as follows:


(2) $$C\,U\,E\%=\frac{A_{T\,O\,C}\times C_{T\,O\,C}\times 10^{6}}{M\times T_{C\,H\,4}}\times 100$$

where the meaning of the designations is the same as that in Equation 1.

#### ^15^N_2_ Fixation (T_N2_)

It includes ^15^N-microbial biomass nitrogen (^15^N-MBN) in root system, and ^15^N-inorganic nitrogen in liquid medium released from microbial mineralization of the fixed organic ^15^N. It is calculated as follows:


(3) $$T_{N\,2}=\frac{A_{T\,O\,N}\times C_{T\,O\,N}\times 10^{6}}{M}+\frac{\left(A_{N\,H\,4}\times C_{N\,H\,4}+A_{N\,O\,3}\times C_{N\,O\,3}+A_{N\,O\,2}\times C_{N\,O\,2}\right)\times V_{l}\times 10^{3}}{M\times m}$$

where T_N2_ is expressed as μmol N g^–1^ *d.w.b.*; the designations *A_NH4_, A_NO3,_* and *A*_NO2_ are the atom excess of ^15^N in the inorganic nitrogen, %; the designations *C_NH4_, C_NO3,_* and *C*_NO2_ are the concentrations of the inorganic nitrogen, mg l^–1^; V_l_ is the volume of liquid medium (40 ml, 0.04 l); *M*, molar mass of ^15^N, g mol^–1^; and *m*, the dry weight biomass of root pellets, g. ^15^N atom abundance was not determined if the bulk concentration of inorganic N was below detection limit (Supplementary Table 1).

#### Methanotrophic N_2_-Fixing Efficiency

Methanotrophic N_2_-fixing efficiency was defined as the amount of ^15^N_2_ fixed when one mole of methane was oxidized, calculated as the ratio of T_CH4_ to T_N2_. This value was employed to make a rough estimate of methane oxidation-induced N_2_ fixation budget in paddy field globally.

### DNA-Stable Isotope Probing and High-Throughput Sequencing

#### DNA Extraction

For day 0 and incubated root samples, approximately 0.2 g of the ground powders were used for bacterial genome DNA extraction using FastDNA Spin Kit for Soil (MP Bi) according to the instructions of the kit. The extracted DNA was dissolved in 50 μl deionized sterile water, and its concentration and quality were determined by a UV spectrophotometer (NanoDrop ND-1000). The obtained DNA extracts were 10–50 ng μl^–1^ with A260/A280 in the range of 1.70–1.85.

#### DNA-SIP

The DNA obtained above were applied to ultra-high-speed density gradient centrifugation according to Jia and Conrad (2009), Xia et al. (2011). Brief steps are 2.0 μg of DNA was mixed with CsCl to form a centrifuge solution with an initial buoyancy density of 1.725 g ml^–1^, which was then transferred to a 5.1 ml centrifuge tube, and placed in a Beckman ultra-high-speed centrifuge for centrifugation at 45,000 rpm at 20°C for 44 h. Centrifuged solutions were divided into 15 fractions with different buoyancy density and collected into 1.5 ml sterile Eppendorf tubes. Each fraction was washed with PEG-6000 to remove CsCl as well as further washed with 70% ethanol to get clean DNA. Finally, the DNA was dissolved in 30 μl sterile water, stored at –20°C for subsequent ^13^C-DNA identification through quantitative polymerase chain reaction (PCR) of *pmoA* and *nifH* genes.

#### PCR and Illumina Sequencing

Bacterial primer sets of 515F/907R (Stubner, 2002) for 16S rRNA gene, A189f/mb661r (Costello and Lidstrom, 1999) for *pmoA* gene, and polF/polR (Poly et al., 2001) for *nifH* gene were used to investigate the total microbial communities, methane-oxidizing, and nitrogen-fixing microbes, respectively. The changes in gene abundances among different treatments as well as the different fractions of isotope labeled DNA were analyzed by quantitative PCR. Quantitative PCR reaction system includes 10 μl of SYBR Premix Ex Taq (Takara), 0.5 μl of primers (10 μM), 1 μl of DNA templates, and 8 μl of sterile water. The amplification curve and melting curve analysis were also performed after PCR cycling procedures for each reaction. All amplifications were conducted in triplicate, with amplification efficiencies in the range of 80–100%, *R*^2^ in 0.990–0.999, using CFX96 Optical Real-Time Detection System (Bio-Rad, Laboratories Inc., Hercules, CA, United States). High-throughput sequencing was employed to investigate the microbial community compositions of total DNA and ^13^C-DNA. The PCR products were gel purified by the Agarose Gel DNA Purification Kit (TaKaRa) and then mixed in equimolar ratios for library preparation. Paired-end sequencing (2 × 300 bp) was conducted using the Illumina MiSeq system. The details for primers and PCR conditions are given in Supplementary Table 2.

### Bioinformatics Analyses

#### 16S rRNA and *nifH* Genes

All raw sequences were processed using the Quantitative Insights Into Microbial Ecology pipeline (Caporaso et al., 2010). The quality control procedure removed reads with average quality score < 20 containing mismatched primers and ambiguous bases. Chimeras were eliminated using VSEARCH program (Rognes et al., 2016). The high-quality sequences were then clustered into operational taxonomic units (OTUs) at 97% similarities for 16S rRNA gene (Edgar, 2010) or 95% for *nifH* gene. For 16S rRNA gene, 1,282,215 clean reads were obtained for all of the 18 DNA samples. SILVA database (version 1.9.5) was used for taxonomic affiliation (Quast et al., 2013). Principal component analysis (PCA) of 16S rRNA gene community was conducted in Canoco5.^1^ The alpha diversity indices were calculated using the function ‘‘diversity’’ in R package ‘‘vegan.’’ For *nifH* gene, online Functional Gene Pipeline/Repository (FunGene^2^) was used for taxonomic analysis. The sequences of representative OTUs were then selected for phylogenetic tree construction with the MEGA5.2 software with bootstrapping of 1,000 replicates (Tamura et al., 2011).

#### *pmoA* Gene

Raw sequence files were processed using the Mothur software (version 1.33.3). The commands “trim.seqs” (minlength = 400 and qaverage = 30) and “split groups” were used for quality control and sample splitting (Schloss et al., 2009). These reads were then processed using the online version of FunGene Pipeline (Fish et al., 2013) to check the chimera using VSEARCH (Rognes et al., 2016) and to correct frameshifts using FramBot (Wang et al., 2013). OTU clustering based on amino acid sequences was performed using the UCLUST algorithm (Edgar, 2010) at a 7% cutoff value (Degelmann et al., 2010). Taxonomic affiliation was performed using the Bayesian method based on a database containing 6,628 *pmoA* and *pmoA*-related sequences of pure culture and uncultured methanotrophic ecotypes (Dumont et al., 2014).

#### Co-occurrence Network Analysis

Network structures of 16S rRNA gene sequences for all of the total DNA, including day 0 and incubated samples, were calculated in SpiecEasi R package. SpiecEasi (Sparse InversE Covariance estimation for Ecological Association and Statistical Inference) is a novel statistical method developed specifically for compositional data and network inference; it is done under the assumption of sparsity using sparse neighborhood and inverse covariance selection algorithms (Kurtz et al., 2015). The resulting adjacency matrices were converted into network objects using the R igraph package. Cytoscape (v 3.8.2) was used for visualizing the networks and identifying network modules (Shannon et al., 2003). Two topological parameters were used to estimate the role of the nodes, namely, within-module connectivity Zi and the among-module connectivity Pi (Guimera and Nunes Amaral, 2005). Module hubs were critical to their own module coherence (Zi > 2.5, Pi ≤ 0.62), connectors connected modules together and were important to network coherence (Zi ≤ 2.5, Pi > 0.62), and network hubs were vital to both the network and their own module coherence (Zi > 2.5, Pi > 0.62), whereas others are defined as peripherals (Zi ≤ 2.5, Pi ≤ 0.62) (Olesen et al., 2007). Module abundance was calculated as the sum of relative abundance of the microbial taxa (OTU) that belonged to it under different treatments. The relationships between relative abundance of each ecological cluster and C, N flow were analyzed using Pearson’s correlation test.

### Statistical Analysis

Different forms of carbon and nitrogen contents and gene copy numbers were presented as mean ± standard deviation of triplicate values for each treatment. Statistical analyses were performed using IBM SPSS Statistics 19.0 (SPSS Inc., Cary, NC, United States). We tested significant differences by a one-way ANOVA with the least significant difference test and a *p*-value of less than 0.05 was considered statistically significant.

## Results

### Microbial Carbon Metabolism and N_2_ Fixation in CH_4_-Consuming Rice Root

**FIGURE 1:**
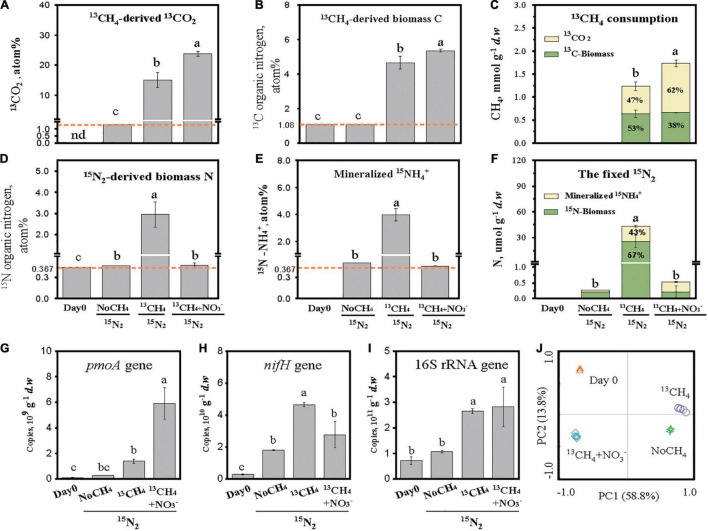
CH_4_-derived carbon flow and N_2_ fixation in CH_4_-consuming rice roots. The designations “NoCH_4,_” “^13^CH_4,_” and “^13^CH_4_+ NO_3_^–^” refer to the microcosms incubated without CH_4_, with 10% (v/v) ^13^CH_4_, and with 10% (v/v) ^13^CH_4_ and KNO_3_ fertilization, respectively. The atom percent of stable isotope C and N is shown as ^13^CH_4_-derived ^13^CO_2_ **(A)**, ^13^CH_4_-derived biomass ^13^C **(B)**, ^13^CH_4_ consumption as the sum of ^13^CH_4_-derived ^13^CO_2_ and ^13^CH_4_-derived biomass ^13^C **(C)**,^15^N_2_-derived biomass ^15^N **(D)**, mineralized ^15^N-NH_4_^+^ from the fixed organic ^15^N **(E)**, and the fixed ^15^N_2_ as the sum of ^15^N_2_-derived biomass ^15^N and mineralized ^15^N-NH_4_^+^ **(F)**. ^13^CO_2_ was calculated on the basis of the headspace CO_2_ concentration and its ^13^C atom percent. ^13^C-MBC was calculated on the basis of the total undissolved organic carbon content in root and its ^13^C atom percent. ^15^N-microbial biomass nitrogen (MBN) was calculated on the basis of the total undissolved organic nitrogen content in root and its ^15^N atom percent. The ^15^NH_4_^+^ represents the ammonium released from mineralization of ^15^N-cellular materials from ^15^N_2_-fixers and was calculated on the basis of the NH_4_^+^ concentration and its ^15^N atom percent. The abundance of methane-oxidizing bacteria, N_2_ fixers, and total root-associated microorganisms was determined by real-time quantitative PCR of the biomarker genes *pmoA* **(G)**, *nifH* **(H)**, and 16S rRNA **(I)**, respectively. Principal component analysis (PCA) plots of 16S rRNA gene sequences were used to assess community shifts of total root-associated microorganisms in rice roots **(J)**. The error bars represent the standard deviations of three microcosms. Different letters above the columns indicate a significant difference among different treatments (*p* < 0.05). n.d., not detected.

The atom percent of ^13^CO_2_ increased from the background level of 1.08% in the absence of CH_4_ (NoCH_4_) to 15.05 and 23.78% in the presence of ^13^CH_4_ and ^13^CH_4_+NO_3_^–^, respectively (Figure 1A). Biomass synthesis of ^13^CH_4_-C resulted in significant enrichment of ^13^C-TOC from 1.08% under the NoCH_4_ treatment to 4.65 and 5.36% under the ^13^CH_4_ and ^13^CH_4_+NO_3_^–^ treatments, respectively (Figure 1B). It indicated that MOB-assimilated ^13^C-CH_4_ contributed 3.57% of the ROC. The net CH_4_ consumption was assessed as the sum of ^13^CO_2_ flux and root ^13^C-TOC content with 1,238 and 1,734 μmol g^–1^ *d.w.b.* under the ^13^CH_4_ and ^13^CH_4_+NO_3_^–^ treatments, respectively (Figure 1C). The carbon utilization efficiencies were 53 and 38% under the ^13^CH_4_ and ^13^CH_4_+NO_3_^–^ treatments, respectively (Figure 1C).

N_2_ fixation was significantly stimulated by methane oxidation as the atom percent of ^15^N-TON in rice roots increased drastically from the background level of 0.369% to the 2.951% in ^13^CH_4_ treatment (Figure 1D). In the absence of methane (NoCH_4_), the atom percent of ^15^N-TON remained unchanged, whereas nitrate amendment (^13^CH_4_+NO_3_^–^) almost abolished completely ^15^N enrichment in TON at 0.385% (Figure 1D). Intriguingly, ^15^N-NH_4_^+^ increased drastically from the background level of 0.369% to the 3.98% in ^13^CH_4_ treatment (Figure 1E), indicating rapid mineralization of ^15^N-TON. After 30 days incubation, the ^15^N-TON contents were 0.204, 24.9, and 0.210 μmol g^–1^ *d.w.b.* under the NoCH_4_, ^13^CH_4_, and ^13^CH_4_+NO_3_^–^ treatments, respectively, and the ^15^N-NH_4_ contents were 0.06, 18.4, and 0.32 μmol g^–1^ *d.w.b.*, respectively (Figure 1F). The fixed N was thus 164-fold higher under ^13^CH_4_ than under NoCH_4_ treatment. Moreover, 42.5% of the fixed N was microbially mineralized as bioavailable ^15^NH_4_^+^ in CH_4_-consuming rice roots (Figure 1F).

The population sizes of MOB and N_2_ fixers were assessed by real-time quantitative PCR analysis of functional *pmoA* and *nifH* gene biomarkers. Being consistent with CH_4_ consumption (Figure 1C) and N_2_ fixation (Figure 1F), a significant increase was observed for the *pmoA* gene (16-fold) and *nifH* gene (20-fold) under ^13^CH_4_ incubation (Figures 1G,H). Nonetheless, ^13^CH_4_+NO_3_^–^ had the highest *pmoA* gene copies, whereas the *nifH* gene abundance was lower than that under the ^13^CH_4_ treatment, suggesting selective growth of MOB carrying no *nifH* genes under ^13^CH_4_+NO_3_^–^. The 16S rRNA gene abundance of the total microbial communities was also stimulated during methane oxidation regardless of nitrate amendment (Figure 1I), and obvious shifts in community structure were observed among the different treatments by PCA (Figure 1J).

### Methane Oxidation as an Engine Driving Carbon and Nitrogen Flows in Rice Root

**FIGURE 2:**
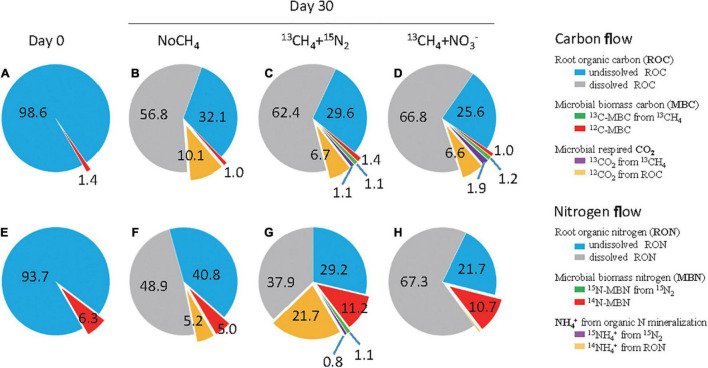
Methanotrophy-directed flow of carbon and nitrogen by rice root-associated microbes. The carbon and nitrogen percentage in rice roots at day 0 **(A,E)** and under three treatments of NoCH_4_ **(B,F)**, ^13^CH_4_ **(C,G)**, and ^13^CH_4_+NO_3_^–^ **(D,H)** at day 30. Briefly, the undissolved root organic carbon (ROC) and nitrogen (RON) were determined on the basis of dry weight. The ^12^C-MBC was estimated by real-time quantification of microbial cells assuming that each cell has the dry weight of 6.2 × 10^– 13^ g (Knief and Dunfield, 2005) and contains 3.82 copies of 16S rRNA genes (Sun et al., 2013). ^12^CO_2_ from ROC was calculated by the difference between the total headspace CO_2_ and the ^13^CO_2_. The ^14^N-MBN was estimated by real-time quantification of microbial cells in a way similar to ^12^C-MBC. The ^14^NH_4_^+^ from RON mineralization was determined by the difference between the total NH_4_^+^ content and ^15^NH_4_^+^ in aqueous phase. The descriptions for other designations are the same as that in Figure 1.

On day 0, ROC and MBC were considered undissolved and accounted for 98.6 and 1.4% of TOC, respectively (Figure 2A). Remarkable decomposition of ROC occurred during 30 days incubation, and the majority was recovered at day 30 as the dissolved ROC with 56.8, 62.4, and 66.8% of TOC under NoCH_4_, CH_4,_ and CH_4_+NO_3_^–^ treatments, respectively (Figures 2B–D). Root-associated methane oxidation triggered 2.5–6.5% more mineralization of ROC than the NoCH_4_ control (Figures 2B–D). Meanwhile, ROC-respired CO_2_ accounted for 6.6–10.1% of TOC, whereas ^13^CO_2_ from methane oxidation was below 1.9%. Intriguingly, root-associated MBC remained largely unchanged from 1.0 to 1.4% of TOC, whereas ^13^CH_4_-derived MBC accounted for 1.1 and 1.2% under CH_4_ and CH_4_+NO_3_^–^ treatments, respectively (Figures 2C,D). It thus indicated strong stimulations of methanotrophy-directed ROC decomposition.

Similarly, rapid mineralization of the undissolved root organic nitrogen (RON) was catalyzed by methanotrophy-mediated microbial communities, leading to a significant decline in RON from 93.7% of TON on day 0 (Figure 2E) to 40.8% (NoCH_4_), 29.2% (^13^CH_4_), and 21.7% (^13^CH_4_+NO_3_^–^) on day 30 (Figures 2F–H). The resulting NH_4_^+^-N content was 3.63, 15.5, and 0.26 μmol N bottle^–1^ under the NoCH_4_, ^13^CH_4_, and ^13^CH_4_+NO_3_^–^ treatments, (Supplementary Table 1), and accounted for 5.2, 21.7, and 0.36% of TON, respectively (Figures 2F–H). The inorganic N was predominated by RON-mineralized ^14^NH_4_^+^-N with approximately 15.0 μmol N bottle^–1^, and methanotrophy-induced fixation of ^15^N_2_ was only 0.55 μmol N bottle^–1^ (Supplementary Table 1). Notably, MBN was also stimulated significantly from 6.3% of TON on day 0 to 11.2 and 10.7% on day 30 under ^13^CH_4_ and ^13^CH_4_+NO_3_^–^ treatments, respectively, whereas CH_4_-induced ^15^N-MBN remained to be as low as 1.1% of TON. In contrast, nitrate amendment completely abolished mineralization of RON to inorganic ^14^NH_4_^+^-N (Figure 2H).

### Active Methanotrophs in CH_4_-Consuming Rice Root

High-throughput sequencing of 16S rRNA gene indicated that more diverse species were induced during methane oxidation and nitrogen fixation. The Shannon index was higher after incubation than that in the fresh roots while the Simpson index displayed an opposite result (Supplementary Table 3). Intriguingly, the relative abundance of methanotrophs remained at a relatively low level, although a significant increase occurred from 0.05 to 0.87% after incubation (Supplementary Table 3).

**FIGURE 3:**
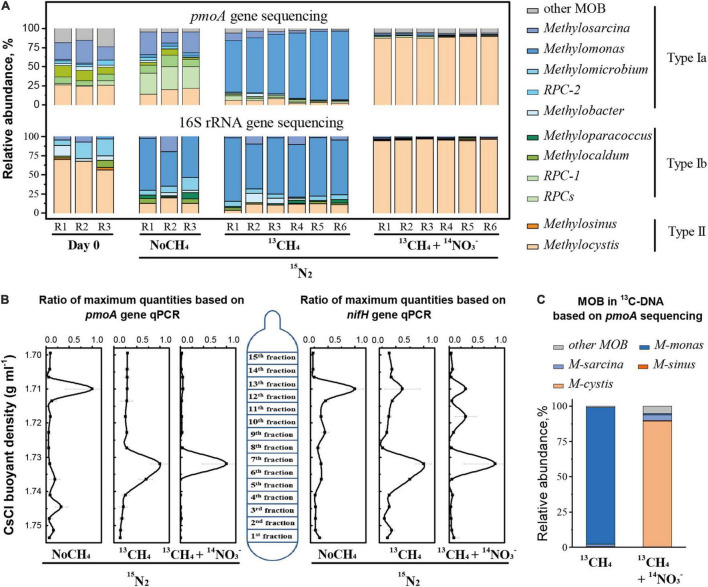
Population dynamics of active methanotrophs in CH_4_-consuming rice roots. Methanotrophic communities in the roots over a 30-day incubation period under different treatments on the basis of *pmoA* and 16S rRNA gene sequencing **(A)**. Abundance of the *pmoA* and *nifH* genes as a function of buoyancy density of the fractionated DNA following ultracentrifugation of the total DNA **(B)**. Taxonomic identities and relative abundance of ^13^C-labeled active methane-oxidizing bacteria (MOB) in ^13^C-DNA **(C)**. Designations “R1–R6” represent biological duplication. Error bars represent the standard deviations of three technical duplicates. The designations “NoCH_4,_” “^13^CH_4,_” and “^13^CH_4_+ NO_3_^–^” are the same as those in Figure 1.

High-throughput sequencing of *pmoA* gene amplicons revealed significant changes in MOB community structure in rice roots (Figure 3A). Over the incubation period, type I *Methylomonas*-like MOB increased drastically from 1.2 to 80% under ^13^CH_4_, whereas type II *Methylocystis*-like MOB increased from 25.3 to 88.6% under ^13^CH_4_^+^NO_3_^–^ treatment (Figure 3A). Similar results were obtained for 16S rRNA gene analysis (Figure 3A). Notably, in NoCH_4_ treatment, MOB members within the uncultured rice paddy cluster (RPC-1 group) showed a significant increase in *pmoA* genes from 2.8 to 28.6%, whereas 16S rRNA genes indicated apparent stimulation of *Methylomonas*-like MOB.

DNA-SIP relying on cell propagation provided further support. The ^13^C-labeled MOB were resolved by real-time quantitative PCR analysis of the *pmoA* and *nifH* genes as a function of the buoyant density of the DNA gradient following ultracentrifugation of the total DNA extracted from rice roots (Figure 3B). A single peak of *pmoA* genes occurred in the ^13^C-labeled “heavy” DNA (fractions 6–7) under ^13^CH_4_, whereas the unlabeled peak of *pmoA* genes remained in the “light” DNA under NoCH_4_. The peak was visualized clearly in the ^13^C-labeled “heavy” fraction DNA (seventh fraction) under ^13^CH_4_+NO_3_^–^ (Figure 3B). Similar patterns were observed for *nifH* gene abundance as a function of DNA buoyant density. High-throughput sequencing of ^13^C-*pmoA* genes further indicated that active MOB were phylogenetically affiliated with *Methylomonas* and *Methylocystis* under the ^13^CH_4_ and ^13^CH_4_+NO_3_^–^ treatments, respectively (Figure 3C).

### Keystone Species Associated With Carbon and Nitrogen Turnover in Rice Root

**FIGURE 4:**
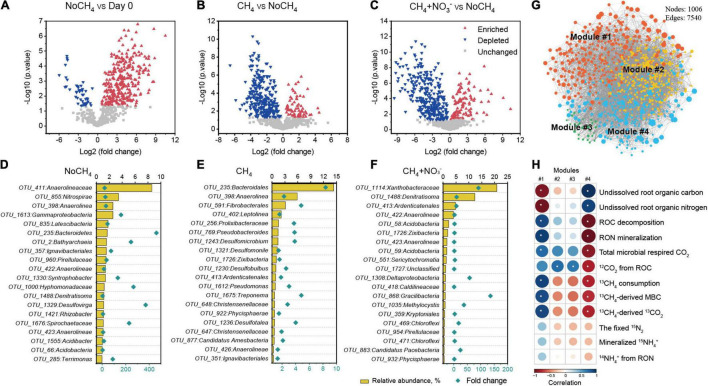
Population dynamics of microbial communities during methanotrophy-directed carbon and nitrogen turnover in rice root. Volcano plot shows significantly changed microbial taxa (operational taxonomic unit, OTU) over a 30-day incubation period for pairwise comparison of root microcosms including NoCH_4_ on day 30 vs. day 0 fresh roots **(A)**, CH_4_ vs. NoCH_4_ on day 30 **(B)**, and CH_4_+NO_3_^–^ vs. NoCH_4_ on day 30 **(C)**. The significantly changed OTUs satisfying the criteria of log 2 (fold-change) value > 0 or < 0 and *p* < 0.05 are considered enriched (red triangle) or depleted (blue triangle), respectively. The unchanged OTUs are represented by gray squares. The top 20 most abundant OTUs are shown, indicating the significantly enriched taxa under treatments of NoCH_4_ **(D)**, CH_4_ **(E)**, and CH_4_+ NO_3_^–^ **(F)**. In each figure, the relative abundances of the OTUs are shown as column while the fold changes are displayed as diamonds. The detailed taxonomy information of the OTUs is listed in Supplementary Table 4. Network diagram is constructed with the colored nodes representing four ecological modules **(G)**, and the size of the node corresponds to the number of connections (degree). Heatmap showing the Person’s correlation between carbon or nitrogen flows and relative abundance of the main ecological clusters **(H)**. Correlations (*r* values) are indicated with gradient colors ranging from red (-1) to blue (1), *p* < 0.05 is marked on the map with stars. The designations in the heatmap were the same as that in Figure 1 or Figure 2, while “ROC decomposition” is the sum of dissolved ROC and ^12^CO_2_ from ROC. “RON mineralization” is the sum of dissolved RON and ^14^NH_4_^+^ from RON.

After incubation for 30 days, statistical analysis showed that 314 root-associated OTUs significantly increased under NoCH_4_ treatment compared to that of day 0 fresh roots (Figure 4A). Similarly, a total of 65 under ^13^CH_4_ (Figure 4B) and 104 OTUs under ^13^CH_4_+NO_3_^–^ (Figure 4C) showed statistically significant increases relative to NoCH_4_ treatment. As for NoCH_4_ treatment, Anaerolineaceae*-*like OTU emerged as the most abundant phylotype, accounting for 8.6% of the total microbial abundance, while Bacteroidete*-*like OTU exhibited the highest 461-fold increase from 0.004 to 1.7% (Figure 4D, Supplementary Table 4). As for ^13^CH_4_ treatment, Bacteroidete-like OTU predominated root-associated microbial communities with 14.3% of total microbial abundance and showed the most significant increase of 8.4-fold (Figure 4E and Supplementary Table 4). As for ^13^CH_4_+NO_3_^–^ treatment, Xanthobacteraceae dominated root-associated microbial communities (20.9% of total microbial abundance) and exhibited the 91-fold increase (Figure 4F and Supplementary Table 4).

Co-occurring pattern analysis of total microbial community further suggested these significantly increased taxa could have played a major role in root organic matter decomposition with rapid growth rates and reproduction ability (Figure 4G). Four ecological clusters (modules) strongly co-occurring with each other (modules #1, #2, #3, and #4) during root methane oxidation. These four modules were characterized by phylogenetically distinct keystone taxa (Supplementary Figure 1). For instance, module #1 contained *Anaerovorax* and the unclassified Gammaproteobacteria, whereas module #2 was defined by Lokiarchaeia, *Galbitalea*, and Chloroflexi (Supplementary Figure 1). Methane oxidation resulted in significant changes in modules. The abundance of module #1 was significantly stimulated for all NoCH_4_, CH_4,_ and CH_4_+NO_3_^–^ treatments, whereas module #4 showed decreasing trends (Supplementary Figure 2). Modules #2 and #3 were more abundant in NoCH_4_ treatment.

Significant correlations between C and N flows and modules suggested a methanotrophy-primed assembly of distinct communities. The abundance of module #1 was negatively correlated with the undissolved ROC and RON, but positively associated with root decomposition, including ROC decomposition, RON mineralization, total CO_2_ respiration, and RON-derived ^14^NH_4_^+^ production. Moreover, there was a positive relationship between module #1 and methanotrophy, including methane consumption, ^13^C-MBC production, and ^13^CO_2_ evolution. Intriguingly, the opposite trend was observed for module #4. Meanwhile, modules #2 and 3 were positively correlated with ROC-derived CO_2_ (Figure 4H).

## Discussion

Our results provide compelling evidence that methanotrophy-directed carbon and nitrogen turnover in rice roots could have played a pivotal role in the self-sustaining productivity of paddy soil. MOB-mediated ^13^C-CH_4_ flow contributed 3.57% of the rice ROC (Figure 1B), being reasonably similar to 10% of *Sphagnum moss* carbon (Raghoebarsing et al., 2005). It also induced strong N_2_-fixing activity and contributed 2.58% of the RON under nitrogen-deplete condition (Figure 1D). Ecophysiology flux and DNA-SIP analysis indicated that methanotrophy-primed MOB and other strongly related taxa could have played a key role in root decomposition and N_2_ fixation to facilitate nutrients cycling while maintaining soil productivity.

It was shown in this study that methane oxidation stimulated biological nitrogen fixation while promoting microbial mineralization of rice RON under nitrogen constraint. A 50-year assessment of the global nitrogen budget for rice production systems indicated that 24% of rice N resulted from nonsymbiotic nitrogen fixation (Ladha et al., 2016). It has been estimated that annual net methane emission during the rice-growing season is 150 kg ha^–1^ (Sun et al., 2020), and up to 90% of methane has been recycled through methane oxidization before being released into the atmosphere (Conrad, 2009). Methanotrophy-induced N_2_ fixation was thus roughly estimated to be 40.7 kg N ha^–1^ in this study, which was close to 45 kg N ha^–1^ under near *in situ* field conditions (Bei et al., 2013). Compared to a C/N ratio of approximately 5 in a previous study in peatland (Larmola et al., 2014), the higher C/N ratio of 29 in the rice root triggered N_2_ fixation of 43.3 μmol N g^–1^ dry weight root biomass by gammaproteobacterial *Methylomonas*-like MOB during methane oxidation. MOB were widely regarded as the biofilters of greenhouse gas CH_4_ in flooded paddies or wetlands, and we provide an experimental evidence for great nitrogen-fixing potential of MOB in this study. The contribution of methanotrophy to nitrogen input deserves more attention in paddy fertilization management. Moreover, up to 42.5% of the newly fixed N existed in the form of ^15^N-NH_4_^+^, suggesting rapid microbial biomass N mineralization under methane oxidation. Therefore, methanotrophy could improve the rice self-sustaining system by providing more inorganic nitrogen that is directly used by plants, especially in the root or rhizosphere with high methane-producing and -oxidizing activities. These results linked the methane oxidation to crop productivity more closely in paddy fields.

A large amount of NH_4_^+^ was released though root decomposition under methane oxidation, which promoted the ratio of NH_4_^+^ mineralization from 5.2 (NoCH_4_) to 21.7% under nitrogen-deplete conditions (Figure 2B). This might be explained by nitrogen mining hypothesis between plant and microbial co-evolution. For example, plants have to be adapted to environment through interaction with soil microorganisms with defined ecosystem C:N:P stoichiometry (Chen and Chen, 2021). Microorganisms are supposed to have much stronger functional flexibility to acquire nitrogen from external environment to alleviate stoichiometric N constraints under excess C condition (Zechmeister-Boltenstern et al., 2015). It was confirmed by our results that microbial N_2_-fixing activity was stimulated under methane oxidation with low nitrogen condition. Besides, excessive carbon input would have intensified nitrogen recycling of microbial necromass (Kaiser et al., 2014; Cui et al., 2020). MOB, as biological engine of rice root decomposition, were likely to stimulate the flow of microbial carbon and nitrogen at different trophic levels, promoting the nutrient recycle and release. In fact, up to 42.5% of the newly fixed organic ^15^N was re-mineralized into NH_4_^+^ (Figure 1F). Moreover, decreasing microbial carbon use efficiency of community metabolism could also be a speculative feedback of nitrogen deficiency (Manzoni et al., 2012). Similarly in this study, 10.1% of ROC was converted into CO_2_ through microbial respiration under NoCH_4_ condition, whereas it was significantly reduced due to methanotrophy-induced N_2_ fixation or nitrate addition (Figure 2A). According to the law of ecological stoichiometry during organic matter degradation, the decomposition rate of organic carbon was higher than that of organic nitrogen, resulting in progressively reduced C:N ratio, and net N loss occurred when C:N ratio was 33–68 with variations across different litter types (Moore et al., 2011). Meanwhile, nitrogen release was found dominantly driven by initial tissue N content, where 1.02–1.98% of nitrogen content was supposed to exhibit nitrogen release (Parton et al., 2007). In this study, rice root with a relatively low C:N ratio of 20 and high N content of 1.73% was used, which may also contribute the nitrogen release during root mineralization.

DNA-SIP revealed a direct link between methane oxidation and taxonomic identities of active microorganisms. It has long been assumed that alphaproteobacterial MOB could be favored under nutrient-poor conditions, whereas gammaproteobacterial MOB rapidly propagates under rich substrate conditions (Murrell and Dalton, 1983). In stark contrast, we observed strong stimulation of *Methylomonas*-like MOB under nitrogen-fixing condition, and *Methylocystis*-like MOB showed preferential growth in nitrate-rich condition. The results might be explained by the rapid degradation or turnover of newly produced methanotrophic cells in the root decomposition system as well as the oxygen condition or other unknown factors in this study. It must be stated that the community catalyzing N_2_ fixation was not exclusively methane oxidizers, although the coupling of methanotrophy-primed carbon flow with N_2_ fixation was clearly demonstrated in this study. *nifH* gene sequencing and networking analysis showed that *Burkholderia* and *Anaeromyxobacter* could also have played a key role in N_2_ fixation, in addition to methanotrophic diazotrophs (Supplementary Figure 3). The contribution of MOB for nitrogen fixation should be delineated by approaches with higher resolutions such as fluorescent *in situ* hybridization (FISH) and Nanoscale Secondary Ion Beam Masspectrometry (NanoSIMS) at single-cell level (Kapili et al., 2020). Multiple samplings at different times would also be of help to elucidate the assembly patterns of active microbial communities in rice paddy field.

Co-occurring pattern analysis indicated that non-methanotrophs played important roles in the C and N turnover. Anaerolineaceae and Bacteroidetes preferred to grow under low nitrogen conditions while an uncultured Xanthobacteraceae taxon was more abundant in nitrate amended condition (Figures 4A–F). Anaerolineaceae belongs to the phylum Chloroflexi, which were predicted to have the ability to degrade diverse plant compounds, such as cellulose, starch, long-chain sugar, and pyrogallol (Hug et al., 2013; Angel et al., 2018). Bacteroidetes were detected in the soil with rice root decomposition (Rui et al., 2009) and was thought to be copiotrophs of *r*-strategy with high growth rate under nutrient-rich conditions (Fierer et al., 2007). These functional guilds could also secrete diverse arrays of carbohydrate-active enzymes for rapid growth (Larsbrink and McKee, 2020). Xanthobacteraceae was one of the very few taxa grown on chemically recalcitrant substrate lignin (Goldfarb et al., 2011). Other specialist taxa might be responsible for the turnover of recalcitrant carbon of root residues, despite their low abundance as slowly growing *K*-strategist (Koch, 2001). Ecological modules provide compelling evidences to elucidate the interactions and organizations of the microbial communities that might be associated with distinct functions (Faust and Raes, 2012). Module #1 showed strong association with the C and N turnover, with its keystone species including *Anaerovorax* and uncultured *Gammaproteobacteria.* These microorganisms are well known for degradation of nitrogen-rich chitin (Dai et al., 2016), implying their strong capability of nitrogen mining. In addition, *Anaerovorax* was known to ferment putrescine to acetate, butyrate, molecular hydrogen, and ammonia (Matthies et al., 2000), and these metabolic products might have been used as substrate to facilitate the growth of more diverse heterotrophs. It seems plausible that *Anaerovorax* served as the keystone taxa driving carbon and nitrogen turnover in this study. It should be noted that a number of uncultured or unclassified phylotypes was revealed in co-occurring pattern, and cultivation-dependent approach would be a key to better understand the couplings of carbon and nitrogen in rice root and soil ecosystems.

## Conclusion

Our findings, based on dual SIP with ^13^CH_4_ and ^15^N_2_, confirmed the methanotrophy-mediated nitrogen fixation in rice root. A large proportion of fixed organic nitrogen was mineralized into the inorganic forms during this process, which could alleviate nitrogen constraint of root carbon turnover. High-throughput sequencing of the microorganisms revealed that *Methylomonas*-like methanotrophs dominated the nitrogen fixation and participated in the root decomposition together with other co-occur groups. Our study indicates that methanotrophy-induced turnover of carbon and nitrogen played an important role in the self-sustaining productivity of paddy soil. It thus provides a starting point for a more sophisticated assay to optimize field management for climate-smart agriculture of rice paddies.

## Data Availability Statement

The datasets presented in this study can be found in online repositories. The names of the repository/repositories and accession number(s) can be found below: https://www.ncbi.nlm.nih.gov/, SRR14456817–SRR14456854 and https://www.ncbi.nlm.nih.gov/, SRR18151151–SRR18151142.

## Author Contributions

ZJ and WC designed the research and analyzed data. WC performed research. YC and ZB helped data mining. SW and XY offered the access to the long-term field experimental site and helped experimental design. WC wrote the first draft. ZJ finalized the manuscript. All authors have seen and approved the final version of the manuscript.

## Conflict of Interest

The authors declare that the research was conducted in the absence of any commercial or financial relationships that could be construed as a potential conflict of interest.

## Publisher’s Note

All claims expressed in this article are solely those of the authors and do not necessarily represent those of their affiliated organizations, or those of the publisher, the editors and the reviewers. Any product that may be evaluated in this article, or claim that may be made by its manufacturer, is not guaranteed or endorsed by the publisher.

## Footnotes

1
http://canoco5.com/

2
http://fungene.cme.msu.edu/
