# Supplementary material for: Methanotrophy Alleviates Nitrogen Constraint of Carbon Turnover by Rice Root-Associated Microbiomes
Source: Front Microbiol. 2022 May 18;13:885087. doi: 10.3389/fmicb.2022.885087 (PMC9159908; doi:10.3389/fmicb.2022.885087)
Supplement: Supplementary file 1 [file Data_Sheet_1.docx]

***Supplementary Materials by Cao et al., 2022***

Methanotrophy alleviates nitrogen constraint of carbon turnover by rice root-associated microbiomes

Weiwei Cao^1,2^, Yuanfeng Cai^1^, Zhihua Bao^3,4^, Shuwei Wang^1^, Xiaoyuan Yan^1^, Zhongjun Jia^1,2*^

*** Correspondence:**

Prof. Dr. Zhongjun Jia

jia@issas.ac.cn

Tel: +86-25-86881311; Fax: +86-25-86881000

**The supplementary materials contain:**

1. Supplementary Table S1

2. Supplementary Table S2

3. Supplementary Table S3

4. Supplementary Table S4

5. Supplementary Figure S1

6. Supplementary Figure S2

7. Supplementary Figure S3

Table S1. Carbon and nitrogen turnover in three different treatments over 30-days incubation, the amounts of different forms of C and N per microcosm bottle were calculated.

| Treatments | | Total carbon, % | Microbial organic C, μmol C bottle^-1^ | Total CO_2_ production, μmol C bottle^-1^ | Net production of ^13^C-CO_2_, μmol C bottle^-1^ | Net production of ^13^C-organic C, μmol C bottle^-1^ | ^13^CH_4_ oxidation, μmol C bottle^-1^ |  | |
| --- | --- | --- | --- | --- | --- | --- | --- | --- | --- |
| Day 0 | Fresh Root | 35.3 ± 0.07a | 23.1 ± 5.32b | n.d. | n.d. | n.d. | n.d. |  | |
| Day 30 | NoCH_4_+^15^N_2_ | 22.1 ± 0.42b | 17.8 ± 0.23b | 165 ± 15.9a | n.d. | n.d. | n.d. |  |  |
|  | ^13^CH_4_+^15^N_2_ | 21.4 ± 1.42b | 42.8 ± 1.29a | 127 ± 4.6b | 17.7 ± 2.83b | 18.9 ± 2.48a | 36.6 ± 4.85b |  |  |
|  | ^13^CH_4_+NO_3_^-^+^15^N_2_ | 18.6 ± 0.49c | 45.7 ± 12.6a | 140 ± 4.8b | 31.8 ± 2.12a | 19.6 ± 0.13a | 51.3 ± 1.98a |  |  |
| Treatments | | Total nitrogen, % | Microbial organic N, μmol N bottle^-1^ | NH_4_^+^ -N, μmol N bottle^-1^ | NO_3_^-^ -N, μmol N bottle^-1^ | NO_2_^-^ -N, μmol N bottle^-1^ | Net production of ^15^N-IN, μmol N bottle^-1^ | Net production of ^15^N-ON, μmol N bottle^-1^ | ^15^N_2_ fixation, μmol N bottle^-1^ |
| Day 0 | Fresh Root | 1.73 ± 0.04a | 4.64 ± 1.07b | n.d. | 410 ± 21.6a | n.d. | n.d. | n.d. | n.d. |
| Day 30 | NoCH_4_+^15^N_2_ | 1.50 ± 0.01b | 3.57 ± 0.05b | 3.63 ± 1.09b | n.d. | n.d. | 0.002 ± 0.001b | 0.01 ± 0.00b | 0.01 ± 0.00b |
|  | ^13^CH_4_+^15^N_2_ | 1.39 ± 0.09b | 8.60 ± 0.26a | 15.5 ± 2.98a | n.d. | n.d. | 0.55 ± 0.04a | 0.74 ± 0.15a | 1.28 ± 0.18a |
|  | ^13^CH_4_+NO_3_^-^+^15^N_2_ | 1.06 ± 0.01c | 9.18 ± 2.54c | 0.26 ± 0.15b | 39.2 ± 7.7b | n.d. | 0.01 ± 0.00b | 0.01 ± 0.01b | 0.02 ± 0.01b |
| Treatments | | C:N ratio | Root apparent C mineralization, μmol C bottle^-1^ | Root-derived CO_2_, μmol C bottle^-1^ | Root-derived DOC, μmol C bottle^-1^ | Root apparent N mineralization, μmol N bottle^-1^ | Root-derived NH_4_^+^-N, μmol N bottle^-1^ | Root-derived DON and gas N, μmol N bottle^-1^ |  |
| Day 0 | Fresh Root | 20.38 ± 0.38a | n.d. | n.d. | n.d. | n.d. | n.d. | n.d. |  |
| Day 30 | NoCH_4_+^15^N_2_ | 14.74 ± 0.42c | 1100 ± 10b | 165 ± 15.8a | 934 ± 5.4c | 37.5 ± 0.30c | 3.63 ± 1.08b | 33.9 ± 1.38b |  |
|  | ^13^CH_4_+^15^N_2_ | 15.81 ± 0.78c | 1135 ± 33b | 110 ± 6.6b | 1026 ± 38.5b | 41.3 ± 1.93b | 15.0 ± 2.95a | 26.3 ± 4.84b |  |
|  | ^13^CH_4_+NO_3_^-^+^15^N_2_ | 17.50 ± 0.23b | 1207 ± 12a | 108 ± 2.6b | 1099 ± 9.4a | 46.8 ± 0.29a | 0.25 ± 0.15b | 46.5 ± 0.44a |  |

Note: n.d: not detected.

*d.w.b*: dry weight biomass, including both the roots and the root-associated microbiomes.

^†^ The detection limit of ammonium, nitrate and nitrite is below 0.046 mg l^-1^, 0.015 mg l^-1^ 0.003 mg l^-1^, respectively.

Table S2. Primers and conditions used in this study.

| Primer | Primer sequence(5'-3') | Target gene | Thermal profile | Molecular analysis |
| --- | --- | --- | --- | --- |
| 515F | GTGCCAGCMGCCGCGG | Bacterial 16S rRNA gene | 95°C, 3min; 39×(95°C, 30s; 55°C, 30s; 72°C, 30s with plate read); Melt curve 65.0 to 95.0°C, increment 0.5°C, 5s + plate read | Real-time quantitative PCR |
| 907R | CCGTCAATTCMTTTRAGTTT |  | 94°C, 5.0 min; 28 cycles (95°C, 30 s; 55°C, 30 s; 72°C, 30 s); 72°C, 10.0 min; hold at 4°C | High throughput sequencing |
| A189f | GGNGACTGGGACTTCTGG | Bacterial *pmoA* gene | 95°C, 3min; 39×(95°C , 10s; 55°C , 30s; 72°C , 30s; 80°C , 5s with plate read); Melt curve 65.0 to 95.0°C, increment 0.5°C, 5s + plate read | Real-time quantitative PCR |
| mb661r | CCGGMGCAACGTCYTTACC |  | 94°C, 5.0 min; 30 cycles (95°C, 30 s; 55°C, 30 s; 72°C, 30 s); 72°C, 10.0 min; hold at 4°C | High throughput sequencing |
| polF | TGCGAYCCSAARGCBGACTC | Bacterial *nifH* gene | 95°C, 30s; 39×(95°C, 10s; 55°C, 30s; 72°C, 30s, 80°C, 5s with plate read); Melt curve 65.0 to 95.0°C, increment 0.5°C, 5s + plate read | Real-time quantitative PCR |
| polR | ATSGCCATCATY TCRCCGGA |  | 94°C, 5.0 min; 30 cycles (95°C, 30 s; 55°C, 30 s; 72°C, 30 s); 72°C, 10.0 min; hold at 4°C | High throughput sequencing |

Table S3. Reads, alpha diversity index and MOB abundance in day 0 and the three treatments, NoCH_4_, ^13^CH_4_ and ^13^CH_4_+NO_3_^-^ in day 30, based on 16S rRNA gene sequencing.

| Treatments | | Reads | Chao1 | Shannon_index | Simpson_index | Goods_coverage | MOB abundance, % |
| --- | --- | --- | --- | --- | --- | --- | --- |
| Day 0 | Fresh Root | 84795 ± 3312a | 813 ± 33b | 1.95 ± 0.13d | 0.327 ± 0.012a | 0.997 ± 0.00a | 0.05 ± 0.01b |
| Day 30 | NoCH_4_+^15^N_2_ | 68280 ± 2648b | 945 ± 35a | 5.04 ± 0.03a | 0.016 ± 0.001d | 0.996 ± 0.00b | 0.15 ± 0.03b |
|  | ^13^CH_4_+^15^N_2_ | 67814 ± 6775b | 846 ± 46b | 4.36 ± 0.08b | 0.041 ± 0.002c | 0.996 ± 0.00b | 0.14 ± 0.02b |
|  | ^13^CH_4_+NO_3_^-^+^15^N_2_ | 69352 ± 8274b | 809 ± 20b | 3.85 ± 0.08c | 0.074 ± 0.009b | 0.996 ± 0.00b | 0.87 ± 012a |

Table S4. Taxonomy information of the top 20 most abundant OTUs enriched in the three treatments, NoCH_4_, ^13^CH_4_ and ^13^CH_4_+NO_3_^-^.

| Taxonomy of the top 20 most abundant OTUs enriched in NoCH_4_ treatment (NoCH_4_ vs Day0) | | | | | |
| --- | --- | --- | --- | --- | --- |
| OTU_ID | Phylum | Class | Order | Family | Genus |
| 411 | Chloroflexi | Anaerolineae | Anaerolineales | Anaerolineaceae | uncultured |
| 855 | Nitrospirae | Thermodesulfovibrionia | uncultured | __ | __ |
| 398 | Chloroflexi | Anaerolineae | Anaerolineales | Anaerolineaceae | Anaerolinea |
| 1613 | Proteobacteria | Gammaproteobacteria | R7C24 | unclassified | unclassified |
| 835 | Latescibacteria | unclassified | unclassified | unclassified | unclassified |
| 235 | Bacteroidetes | Bacteroidia | Bacteroidales | Bacteroidetes vadinHA17 | unclassified |
| 2 | Crenarchaeota | Bathyarchaeia | unclassified | unclassified | unclassified |
| 357 | Bacteroidetes | Ignavibacteria | Ignavibacteriales | SR-FBR-L83 | unclassified |
| 960 | Planctomycetes | Planctomycetacia | Pirellulales | Pirellulaceae | uncultured |
| 422 | Chloroflexi | Anaerolineae | RBG-13-54-9 | unclassified | unclassified |
| 1330 | Proteobacteria | Deltaproteobacteria | Syntrophobacterales | Syntrophobacteraceae | Syntrophobacter |
| 1000 | Proteobacteria | Alphaproteobacteria | Caulobacterales | Hyphomonadaceae | SWB02 |
| 1488 | Proteobacteria | Gammaproteobacteria | Betaproteobacteriales | Rhodocyclaceae | Denitratisoma |
| 1329 | Proteobacteria | Deltaproteobacteria | Syntrophobacterales | Syntrophobacteraceae | Desulfovirga |
| 1421 | Proteobacteria | Gammaproteobacteria | Betaproteobacteriales | Burkholderiaceae | Rhizobacter |
| 1676 | Spirochaetes | Spirochaetia | Spirochaetales | Spirochaetaceae | uncultured |
| 423 | Chloroflexi | Anaerolineae | SBR1031 | unclassified | unclassified |
| 1555 | Proteobacteria | Gammaproteobacteria | Gammaproteobacteria Incertae Sedis | Unknown Family | Acidibacter |
| 66 | Acidobacteria | Subgroup 6 | unclassified | unclassified | unclassified |
| 285 | Bacteroidetes | Bacteroidia | Chitinophagales | Chitinophagaceae | Terrimonas |
| Taxonomy of the top 20 most abundant OTUs enriched in CH_4_ (CH_4_ vs NoCH_4_) | | | | | |
| OTU_ID | Phylum | Class | Order | Family | Genus |
| 235 | Bacteroidetes | Bacteroidia | Bacteroidales | Bacteroidetes vadinHA17 | unclassified |
| 398 | Chloroflexi | Anaerolineae | Anaerolineales | Anaerolineaceae | Anaerolinea |
| 591 | Fibrobacteres | Fibrobacteria | Fibrobacterales | possible family 01 | unclassified |
| 402 | Chloroflexi | Anaerolineae | Anaerolineales | Anaerolineaceae | Leptolinea |
| 256 | Bacteroidetes | Bacteroidia | Bacteroidales | Prolixibacteraceae | uncultured |
| 769 | Firmicutes | Clostridia | Clostridiales | Ruminococcaceae | Pseudobacteroides |
| 1243 | Proteobacteria | Deltaproteobacteria | Desulfovibrionales | Desulfomicrobiaceae | Desulfomicrobium |
| 1321 | Proteobacteria | Deltaproteobacteria | Syntrophobacterales | Syntrophaceae | Desulfomonile |
| 1726 | Zixibacteria | unclassified | unclassified | unclassified | unclassified |
| 1230 | Proteobacteria | Deltaproteobacteria | Desulfobacterales | Desulfobulbaceae | Desulfobulbus |
| 413 | Chloroflexi | Anaerolineae | Ardenticatenales | uncultured | __ |
| 1612 | Proteobacteria | Gammaproteobacteria | Pseudomonadales | Pseudomonadaceae | Pseudomonas |
| 1675 | Spirochaetes | Spirochaetia | Spirochaetales | Spirochaetaceae | Treponema |
| 648 | Firmicutes | Clostridia | Clostridiales | Christensenellaceae | uncultured |
| 922 | Planctomycetes | Phycisphaerae | MSBL9 | SG8-4 | unclassified |
| 1236 | Proteobacteria | Deltaproteobacteria | Desulfobacterales | Desulfobulbaceae | Desulfotalea |
| 647 | Firmicutes | Clostridia | Clostridiales | Christensenellaceae | Christensenellaceae R-7 group |
| 877 | Patescibacteria | Microgenomatia | Candidatus Amesbacteria | unclassified | unclassified |
| 426 | Chloroflexi | Anaerolineae | SJA-15 | unclassified | unclassified |
| 351 | Bacteroidetes | Ignavibacteria | Ignavibacteriales | BSV40 | unclassified |
| Taxonomy of the top 20 most abundant OTUs enriched in CH_4_+NO_3_^-^ treatment (CH_4_+NO_3_^-^ vs NoCH_4_) | | | | | |
| OTU_ID | Phylum | Class | Order | Family | Genus |
| 1114 | Proteobacteria | Alphaproteobacteria | Rhizobiales | Xanthobacteraceae | uncultured |
| 1488 | Proteobacteria | Gammaproteobacteria | Betaproteobacteriales | Rhodocyclaceae | Denitratisoma |
| 413 | Chloroflexi | Anaerolineae | Ardenticatenales | uncultured | __ |
| 422 | Chloroflexi | Anaerolineae | RBG-13-54-9 | unclassified | unclassified |
| 58 | Acidobacteria | Subgroup 17 | unclassified | unclassified | unclassified |
| 1726 | Zixibacteria | unclassified | unclassified | unclassified | unclassified |
| 423 | Chloroflexi | Anaerolineae | SBR1031 | unclassified | unclassified |
| 59 | Acidobacteria | Subgroup 18 | unclassified | unclassified | unclassified |
| 551 | Cyanobacteria | Sericytochromatia | unclassified | unclassified | unclassified |
| 1727 | unclassified | unclassified | unclassified | unclassified | unclassified |
| 1308 | Proteobacteria | Deltaproteobacteria | NB1-j | unclassified | unclassified |
| 418 | Chloroflexi | Anaerolineae | Caldilineales | Caldilineaceae | uncultured |
| 868 | Patescibacteria | Gracilibacteria | unclassified | unclassified | unclassified |
| 1035 | Proteobacteria | Alphaproteobacteria | Rhizobiales | Beijerinckiaceae | Methylocystis |
| 359 | Bacteroidetes | Ignavibacteria | Kryptoniales | BSV26 | unclassified |
| 469 | Chloroflexi | OLB14 | unclassified | unclassified | unclassified |
| 954 | Planctomycetes | Planctomycetacia | Pirellulales | Pirellulaceae | Pir2 lineage |
| 471 | Chloroflexi | SHA-26 | unclassified | unclassified | unclassified |
| 883 | Patescibacteria | Microgenomatia | Candidatus Pacebacteria | unclassified | unclassified |
| 932 | Planctomycetes | Phycisphaerae | Pla1 lineage | unclassified | unclassified |


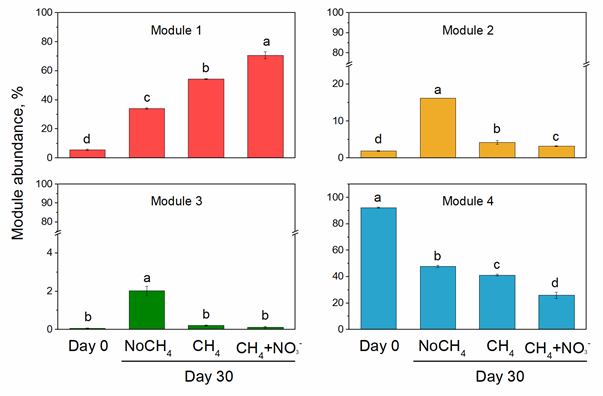
**
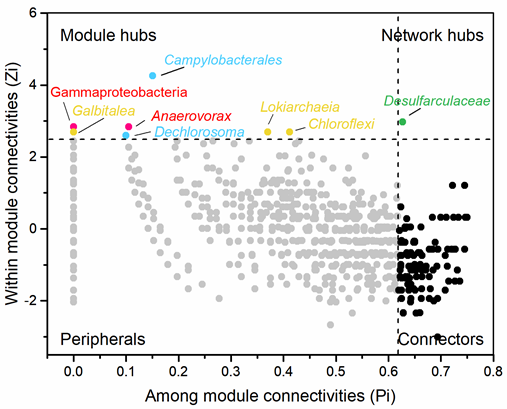
**Figure S1. **Zi-Pi plots showing distribution of OTUs based on their topological roles in co-occurrence network.** Threshold values of Zi and Pi for categorizing OTUs were 2.5 and 0.62 with four groups were sorted including module hubs (Zi>2.5, Pi≤0.62), network hubs (Zi>2.5, Pi>0.62), connectors (Zi≤2.5, Pi>0.62), and the peripherals (zi≤2.5, pi≤0.62). Red, orange and blue labels indicates the keystone taxa of module #1, #2 and #3, respectively, while the green label was the network hub taxa, which was in module #3.

Figure S2. **Relative abundance of the module #1-4 in Day 0 and the three treatments (NoCH_4_, CH_4_ and CH_4_+NO_3_^-^) of Day 30.** Module abundance was defined as the sum of relative abundance of the microbial taxa (Operational Taxonomic Unit, OTU) that belonged to it.


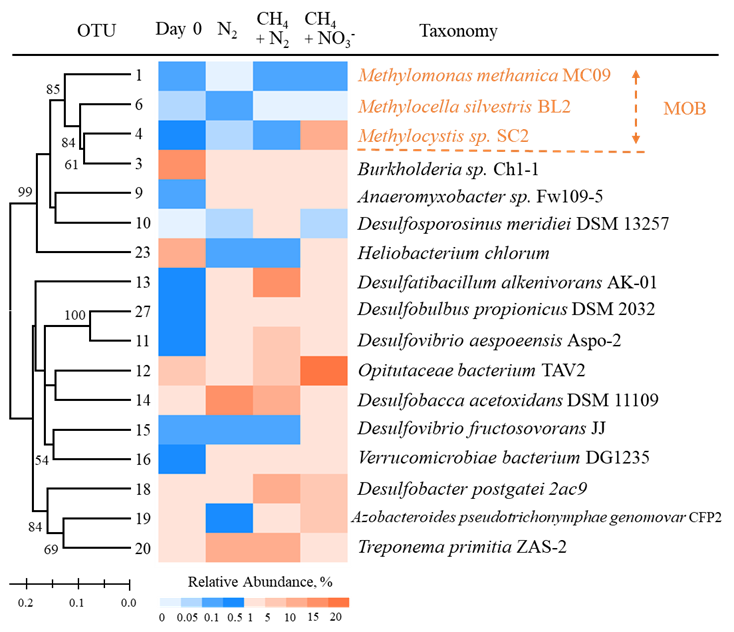
Figure S3. **Phylogenetic tree and heat map for relative abundances of major nitrogen fixing OTUs in the root samples of day 0 and three different treatments based on *nifH* gene sequencing.** The relative abundances of OTUs in each sample are indicated on the heat map with gradient colors ranging from light blue (0%) to orange (20%), and the results of a BLAST search using the representative sequences. The tree was constructed by the neighbor-joining method, and bootstrap values (%) are based on 1,000 replicates. Bootstrap values (>50%) are indicated to the left of nodes in the tree.
